# Neural Progenitor‐Like Cells Induced from Human Gingiva‐Derived Mesenchymal Stem Cells Regulate Myelination of Schwann Cells in Rat Sciatic Nerve Regeneration

**DOI:** 10.5966/sctm.2016-0177

**Published:** 2016-09-07

**Authors:** Qunzhou Zhang, Phuong Nguyen, Qilin Xu, Wonse Park, Sumin Lee, Akihiro Furuhashi, Anh D. Le

**Affiliations:** ^1^ Department of Oral and Maxillofacial Surgery and Pharmacology, University of Pennsylvania School of Dental Medicine, Philadelphia, Pennsylvania, USA; ^2^ Division of Plastic and Reconstructive Surgery, University of Pennsylvania Perelman School of Medicine, Philadelphia, Pennsylvania, USA; ^3^ Department of Oral and Maxillofacial Surgery, Hospital of the University of Pennsylvania, Philadelphia, Pennsylvania, USA

**Keywords:** Peripheral nerve regeneration, Gingiva‐derived mesenchymal stem cell, Induced neural progenitor cells, Schwann cells, Myelination

## Abstract

Regeneration of peripheral nerve injury remains a major clinical challenge. Recently, mesenchymal stem cells (MSCs) have been considered as potential candidates for peripheral nerve regeneration; however, the underlying mechanisms remain elusive. Here, we show that human gingiva‐derived MSCs (GMSCs) could be directly induced into multipotent NPCs (iNPCs) under minimally manipulated conditions without the introduction of exogenous genes. Using a crush‐injury model of rat sciatic nerve, we demonstrate that GMSCs transplanted to the injury site could differentiate into neuronal cells, whereas iNPCs could differentiate into both neuronal and Schwann cells. After crush injury, iNPCs, compared with GMSCs, displayed superior therapeutic effects on axonal regeneration at both the injury site and the distal segment of the injured sciatic nerve. Mechanistically, transplantation of GMSCs, especially iNPCs, significantly attenuated injury‐triggered increase in the expression of c‐Jun, a transcription factor that functions as a major negative regulator of myelination and plays a central role in dedifferentiation/reprogramming of Schwann cells into a progenitor‐like state. Meanwhile, our results also demonstrate that transplantation of GMSCs and iNPCs consistently increased the expression of Krox‐20/EGR2, a transcription factor that governs the expression of myelin proteins and facilitates myelination. Altogether, our findings suggest that transplantation of GMSCs and iNPCs promotes peripheral nerve repair/regeneration, possibly by promoting remyelination of Schwann cells mediated via the regulation of the antagonistic myelination regulators, c‐Jun and Krox‐20/EGR2. Stem Cells Translational Medicine *2017;6:458–470*

**Significance Statement**

Fully functional recovery of injured peripheral nerve remains a major clinical challenge. Stem cell‐based therapy is emerging as a novel paradigm for peripheral nerve regeneration. Neural stem (or progenitor) cells (NSCs) are considered an ideal candidate seed cell source for nerve regeneration, but it remains a challenge to obtain enough transplantable NSCs for clinical application. This report shows that human gingiva‐derived mesenchymal stem cells (GMSCs) can be easily induced into neural progenitor‐like cells (iNPCs). Importantly, the results strongly suggest that GMSCs, particularly GMSC‐derived iNPCs, promote peripheral nerve regeneration, possibly by regulating the expression of the antagonistic myelination regulators c‐Jun and Krox‐20/EGR2 in Schwann cells in rat sciatic nerves.

## Introduction

Injuries to the peripheral nervous system (PNS) are debilitating and usually lead to considerable long‐term disability as a result of loss of nerve and end‐organ target functions 1. Even though the endogenous repair process is initiated after nerve injury, intrinsic regenerative capability is rather limited, especially when there is a large gap between the damaged nerve stumps 2, 3. In the last decade, even though significant effort has been made to develop various types of bioengineered nerve grafts to facilitate peripheral nerve regeneration 2, the overall clinical outcomes still remain suboptimal 4. Recently, there has been growing enthusiasm for the use of stem cell‐oriented tissue engineering technologies to facilitate regeneration of injured peripheral nerves. Among various types of stem cells, NSCs are considered an ideal seed candidate for cell‐based treatment of nerve injury and neurodegenerative diseases; however, it remains a challenge to generate enough transplantable NSCs for clinical application. Alternatively, other types of adult stem cells, particularly mesenchymal stem cells (MSCs) isolated from different tissues such as bone marrow 5, 6, 7, 8, 9, adipose tissue 10, 11, 12, 13, 14, 15, skeletal muscle 16, 17, and dental pulp 18, 19, 20, 21, also confer properties to transdifferentiate into neuronal and glial progenies and produce neurotrophic factors, serving as relevant sources for stem cell‐based therapy in peripheral nerve injury (PNI) 12, 14, 19, 20, 21, 22. However, the high heterogeneity, limited and variable neural differentiation and survival abilities, unpredictable cell fate, and variation of clinical outcomes significantly hinder the clinical application of MSCs in peripheral nerve regeneration.

Expandable and multipotent neural progenitor cells (NPCs) can be generated from embryonic stem cells and induced pluripotent stem cells (iPSCs), but the process is complicated and time‐consuming and raises safety concerns in clinical application 23. Recently, it has been shown that different types of somatic cells could be directly induced into expandable NPCs by overexpressing certain transcriptional factors 24, 25, 26, 27, 28, 29, 30. Interestingly, somatic cells, including fibroblasts and MSCs, can also be directly induced into multipotent NPC‐like cells by small‐molecule compounds or under specific growth conditions without introducing exogenous transcription factors 11, 31, 32, 33, 34, 35. Such novel nongenetic approaches to generate conditionally induced NPC‐like cells may hold great promise for autologous NPC‐based therapy of peripheral nerve regeneration.

Repair of the injured peripheral nerve depends on the plasticity of Schwann cells, the glial cells of the PNS. In response to injury, Schwann cells can dedifferentiate, proliferate, phagocytose myelin debris, and release a myriad of chemokines and cytokines to recruit immune cells to the injured nerve and neurotrophic factors to support neuron survival and promote axon regrowth 36, 37, 38. The reversible transition between dedifferentiation and myelination status of Schwann cells is orchestrated by a complex cross‐antagonistic network of transcriptional regulators, whereby the zinc finger transcription factor Krox‐20/EGR2 governs the expression of a set of myelination‐related genes, and the transcription factor c‐Jun, a negative regulator of myelination, plays a crucial role in the dedifferentiation of Schwann cells 38, 39, 40. The contribution of stem cells to peripheral nerve regeneration may involve their role in Schwann cell transdifferentiation 14, 15 and paracrine neurotrophic and angiogenic effects 12, 20, 22, but little is known about the underlying mechanisms of stem cell‐mediated regulation of dedifferentiation and myelination of Schwann cells in injured peripheral nerves.

We have recently isolated a unique subpopulation of multipotent MSCs from human gingiva (GMSCs) that is of neural crest origin, is highly proliferative, and possesses potent immunomodulatory functions and neurogenic differentiation potential 41, 42, 43, 44. The purpose of the present study is to adopt nongenetic methods to induce multipotent and expandable NPC‐like cells from human GMSCs and explore the feasibility of regenerative stem cell treatment in PNI. Compellingly, GMSC‐derived NPC‐like cells (iNPCs) could differentiate into neuronal and Schwann cells both in vitro and in vivo. More importantly, using a crush‐injury rat sciatic nerve model, we demonstrate that perineural transplantation of iNPCs promotes Schwann cell and axonal regeneration at both the injury and distal segments of the injured nerve. Mechanistically, GMSCs and iNPCs significantly increased the expression of myelination regulatory gene Krox‐20/EGR2 and concomitantly suppressed the expression of dedifferentiation regulatory protein c‐Jun. This study provided the first line of evidence that GMSCs could be transiently converted into multipotent iNPCs under specific culture conditions and that these unique iNPCs are capable of promoting peripheral nerve regeneration, possibly by regulating the dedifferentiation and myelination of Schwann cells.

## Materials and Methods

### Animals

Female Sprague‐Dawley rats weighing 200–250 g (6–7 weeks old) were obtained from Charles River Laboratories (Wilmington, MA, http://www.criver.com). All animal procedures were handled according to the guidelines of the Institutional Animal Care and Use Committee of the University of Pennsylvania. We adopted a randomized, prospective, controlled animal model design according to all the recommendations of the Animal Research: Reporting In Vivo Experiments (ARRIVE) guidelines. Rats were group‐housed in polycarbonate cages (two animals per cage) in the animal facilities with controlled temperature (23°C ± 2°C), 40%–65% humidity, and 12‐hour light/dark cycle. Rats were acclimatized for at least 1 week before the study, fed with a standard laboratory diet, and allowed ad libitum access to drinking water.

### In Vitro Induction of iNPCs From Human GMSCs

Gingival tissues were obtained as remnants of discarded tissues under the approved Institutional Review Board protocol at the University of Pennsylvania. The isolation and characterization of human gingival tissue‐derived MSCs were described previously 41. To induce NPC‐like cells from GMSCs, adherent monolayer GMSCs were trypsinized and seeded onto poly‐l‐ornithine/laminin‐treated 6‐well plates (2 × 10^5^ cells/well) and cultured with complete α‐minimum essential medium for 24 hours. The medium was replaced with neural cell culture medium (neurobasal medium supplemented with 1% N‐2 Supplement, 1% B27, 100 U/ml penicillin, 100 μg/ml streptomycin, and 550 μM 2‐mercaptoethanol; all from Thermo Fisher Scientific Life Sciences, Waltham, MA, http://www.thermofisher.com), 20 ng/ml epidermal growth factor (EGF) and 20 ng/ml basic fibroblast growth factor (bFGF) (PeproTech, Rocky Hill, NJ, http://www.peprotech.com). Under this condition, neurosphere formation was observed at approximately 3–6 days. The neurospheres were completely dissociated into single cells using StemPro Accutase Cell Dissociation Reagent (Thermo Fisher), and cell viability was determined by Trypan blue staining. Dissociated cells were seeded onto polyornithine/laminin‐precoated dishes for further culture.

### In Vitro Neural Differentiation

GMSC‐derived NPC‐like cells at passages 3–4 were seeded on poly‐d‐lysine‐ and laminin‐coated plastic coverslips (Nunc) and cultured in neurobasal medium supplemented with 1% N‐2 Supplement, 5% fetal bovine serum, 0.5 µM all‐*trans*‐retinoic acid (Sigma‐Aldrich, St. Louis, MO, http://www.sigmaaldrich.com), and 10 ng/ml brain‐derived neurotrophic factor (PeproTech) for neuronal differentiation 33. For Schwann cell differentiation, cells were cultured in regular MSC culture medium supplemented with 35 ng/ml all *trans*‐retinoic acid for 72 hours. The medium was changed to regular culture medium supplemented with 5 µM forskolin (Sigma‐Aldrich), 10 ng/ml bFGF, 5 ng/ml platelet‐derived growth factor AA, and 200 ng/ml heregulin‐β‐1 (PeproTech) 14, 19. The medium was replenished every 3 days. After 2 weeks, cells were prepared for immunocytofluorescence studies to examine the expression of neuronal or glial cell markers.

### Immunofluorescence Staining

Cells fixed with 4% paraformaldehyde were blocked and permeabilized for 1 hour at room temperature in PBS, 2.5% goat serum, and 0.5% Triton X‐100, followed by incubation with the following primary antibodies at the appropriate dilution overnight at 4°C: Oct‐4 (mouse IgG, 1:250, sc5279; Santa Cruz Biotechnology, Santa Cruz, CA, http://www.scbt.com), Sox1 (rabbit IgG, 1:250, ab109290; Abcam, Cambridge, MA, http://www.abcam.com), Pax6 (rabbit IgG, 1:250, GTX113241; GeneTex, Irvine, CA, http://www.genetex.com), Nestin (mouse IgG, 1:250, MAB5326; EMD Millipore, Billerica, MA, http://www.emdmillipore.com), Vimentin (mouse IgG, 1:300, sc73528; Santa Cruz Biotechnology), β‐tubulin III (mouse IgG, 1:250, MCA2047; Bio‐Rad, Hercules, CA, http://www.bio‐rad.com), and S‐100β (rabbit IgG, 1:250, ABN59; EMD Millipore). After washing with PBS, cells were incubated with appropriate secondary antibodies at room temperature for 1 hour: goat antirabbit IgG‐fluorescein isothiocyanate (FITC) (1:250, sc2012; Santa Cruz Biotechnology), goat antirabbit IgG‐rhodamine (1:250, sc2091; Santa Cruz Biotechnology), goat antimouse IgG‐FITC (1:250, sc‐2010; Santa Cruz Biotechnology), and goat antimouse IgG‐rhodamine (1:250, sc2092; Santa Cruz Biotechnology). Isotype‐matched control antibodies (BioLegend, San Diego, CA, http://www.biolegend.com) were used as negative controls. Nuclei were counterstained with 4′,6‐diamidino‐2‐phenylindole (DAPI). Images were captured using an Olympus inverted fluorescence microscope (IX73) (Olympus Corporation of the Americas, Center Valley, PA, http://www.olympusamerica.com). For semiquantitative analysis, cells with positive signals in at least six random high‐power fields were visualized, counted, and expressed as the percentage of total DAPI‐positive cells 41. For flow cytometric analysis, intracellular immunostaining of Nestin, Sox‐1, Pax‐6, and Vimentin in cells was performed using nuclear factor fixation, permeabilization, and staining buffer sets according to manufacturer's protocols (BioLegend). After immunostaining, cells were resuspended in 0.5 ml cell staining buffer and analyzed with a FACSCalibur (BD Biosciences, San Jose, CA, http://www.bdbiosciences.com).

### Western Blot Analysis

Cell or sciatic nerve tissue lysates were separated on sodium dodecyl sulfate‐polyacrylamide gel and electroblotted onto nitrocellulose membrane (Bio‐Rad). After blocking with Tris‐buffered saline/5% nonfat dry milk, the membrane was incubated with antibodies against Oct‐4, Sox‐1, Pax‐6, Vimentin, β‐tubulin III, S100‐β, KROX20/EGR2 (Bioss Antibodies, Woburn, MA, http://www.biossusa.com), and c‐Jun (BD Biosciences) followed by incubation with a horseradish peroxidase‐conjugated secondary antibody, and the signals were visualized by enhanced chemiluminescence detection (Santa Cruz Biotechnology). The blots were also reprobed with a specific antibody against β‐actin (Sigma‐Aldrich).

### Enzyme‐Linked Immunosorbent Assay

The concentration of brain‐derived neurotrophic factor, glial‐derived neurotrophic factor (GDNF), nerve growth factor‐β, neurotrophin 3, vascular endothelial growth factor (VEGF), and interleukin‐6 in conditioned media from GMSCs, three‐dimensional (3D)‐spheroids, and iNPCs were detected using enzyme‐linked immunosorbent assay kits according to the manufacturer's protocols (ScienCell Research Laboratories, Carlsbad, CA, http://wwwsciencellonline.com).

### Crush Injury of Rat Sciatic Nerve and Cell Transplantation

Female Sprague‐Dawley rats were anesthetized by intraperitoneal injection of a mixture of ketamine/xylazine (100/10 mg/kg body weight). An incision was made from the right sciatic notch to the distal thigh, and the subcutaneous tissue was bluntly dissected to expose the bicep femoris muscle. The sciatic nerve was exposed and crushed at a point 1 mm distal to the sciatic nerve bifurcation with a type 5 watchmaker forceps for 30 seconds as previously described 14, 45. A combinatorial cell‐scaffold product was developed using PKH26‐prelabeled GMSCs or iNPCs (2 × 10^5^ cells) seeded on GelFoam (6 × 4 × 3 mm). Immediately after nerve crush injury, the cell‐scaffold product was transplanted and wrapped around the injury site of the nerve structure. Rats with sciatic nerve injury and transplanted with GelFoam alone served as control groups. At different time points after nerve injury and cell transplantation, animals were sacrificed and sciatic nerves were harvested for further analysis (supplemental online Fig. 4A).

### Histological and Immunohistochemical Studies

The gastrocnemius muscles of both hindlimbs were harvested 4 weeks after surgery and weighed. The sciatic nerves were fixed in 4% paraformaldehyde for 24 hours, cryoprotected in 10%, 20%, and 30% sucrose, and embedded in optimal cutting temperature medium, and 10‐µm‐thick cryostat sections were cut. After blocking and permeabilization in PBS + 3% bovine serum albumin + 0.5%Triton X‐100 at room temperature for 1 hour, the sections were incubated with primary antibodies for β‐tubulin III (1:250) or S‐100β (1:250) overnight at 4°C, followed by incubation with FITC‐conjugated secondary antibodies for 1 hour at room temperature. Isotype‐matched control antibodies (BioLegend) were used as negative controls. Nuclei were counterstained with DAPI. The integrated immunofluorescence density of a region of interest was quantified using ImageJ (National Institutes of Health), and the corrected total cryosection fluorescence was calculated as average of integrated density − (average of selected areas × mean fluorescence of background), as previously described 46.

### Statistical Analysis

Differences between experimental and control groups were analyzed by paired Student's *t* test. One‐way analysis of variance was used to test the statistical significance of multiple group differences, unless otherwise indicated. Post hoc pairwise comparison between individual groups was made using the Tukey test. *p* values less than .05 were considered statistically significant. SPSS software was used for all the analyses. All data were expressed as mean ± SE.

## Results

### Induction of NSC‐Related Genes in GMSCs

**Figure 1:**
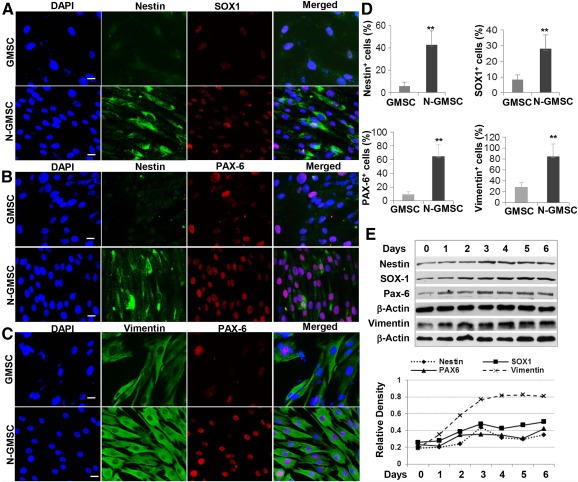
Increased expression of neural stem cell‐related genes in GMSCs cultured in neural medium. GMSCs were cultured in neurobasal medium supplemented with 1% N‐2 Supplement, 2% B27, 20 ng/ml EGF, and 20 ng/ml bFGF for different time periods. **(A–C):** Immunofluorescence studies on the expression of Nestin, SOX1, PAX6, and Vimentin. Nuclei were counterstained with DAPI (blue). Scale bars = 20 µm (×40). **(D):** Percentage of cells positive for each marker. ∗∗, *p* < .01. **(E):** Western blot analysis of the expression of Nestin, SOX1, PAX6, and Vimentin; graph shows densities relative to β‐actin as the internal control. Abbreviations: bFGF, basic fibroblast growth factor; DAPI, 4′,6‐diamidino‐2‐phenylindole; EGF, epidermal growth factor; GMSC, gingiva‐derived mesenchymal stem cell N‐GMSC, neural culture of gingiva‐derived mesenchymal stem cell.

We first examined the expression of NSC‐related genes 33 in adherent GMSCs cultured as a monolayer under neural induction conditions. Immunofluorescence staining showed that exposure of GMSCs to the neurobasal medium supplemented with 1% N‐2 Supplement, 2% B27, 20 ng/ml EGF, and 20 ng/ml bFGF for 3 days significantly upregulated the expression of Nestin, Sox‐1, Pax‐6, and Vimentin compared with regular culture conditions (Fig. 1A–1C). The proportion of NSC‐positive cells, specifically Nestin^+^ cells, increased from 5.74% to 42.7%, Sox‐1^+^ cells increased from 8.44% to 28.06%, Pax‐6^+^ cells increased from 8.98% to 64.64%, and Vimentin^+^ cells increased from 28.88% to 84.6% (Fig. 1D). In addition, Western blot analysis further confirmed a time‐dependent increase in the expression of these NSC‐related genes in GMSCs, which peaked by day 3 under neural culture conditions (Fig. 1E). These results suggest that GMSCs have the potential to be converted into NSC‐like cells under neural induction conditions.

We then determined the expression of NSC‐related genes in GMSCs under 3D‐spheroid culture exposed to neural induction conditions. The cells spontaneously aggregated into 3D‐spheroid structures with positive 5‐bromo‐2′‐deoxyuridine incorporation, suggesting their proliferating status (supplemental online Fig. 1A). Immunostaining showed elevated expression of NSC‐related genes, such as Nestin, Sox‐1, Pax‐6, and Vimentin (supplemental online Fig. 1B–1D). Quantitatively, flow cytometric analysis of 3D‐spheroid GMSCs confirmed the enhanced expression of neural differentiation markers, specifically a markedly increase in the proportion of Nestin^+^ cells from 3.2% to 37.8%, Sox‐1^+^ cells from 5.6% to 22.4%, and Pax‐6^+^ cells from 2.7% to 30.8%, compared with the regular adherent GMSCs (supplemental online Fig. 1E). The increased expression of NSC‐related genes in spheroid GMSCs was further confirmed by Western blot analysis (supplemental online Fig. 1F), showing the time‐dependent expression of these gene products. These results suggest that 3D‐spheroid culture can enhance NSC‐related gene expressions in GMSCs.

### Induction of NPCs From GMSCs

**Figure 2:**
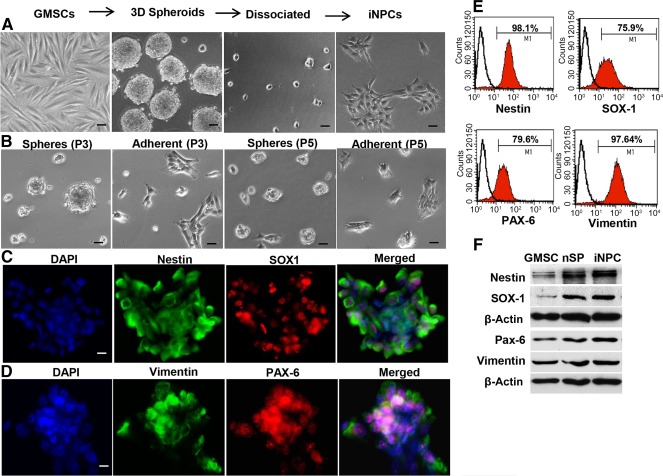
Conditional induction of GMSCs into iNPCs. GMSCs were cultured in neurobasal medium supplemented with 1% N‐2 Supplement, 2% B27, 20 ng/ml EGF, and 20 ng/ml bFGF under suspension culture conditions for 3 days. **(A):** Spheroid cells were dissociated and cultured in polyornithine/laminin‐coated 6‐well plates with the same medium for 7 days. Morphological changes were observed under a microscope. **(B):** Subculture of GMSC‐derived spheroid and iNPCs. Scale bars = 100 µm (×10) **(C,D):** Immunofluorescence studies on the expression of Nestin, SOX1, PAX6, and Vimentin in GMSC‐derived iNPC colonies. Nuclei were counterstained with DAPI (blue). Scale bars = 20 µm (×40). **(E):** Percentage of cells positive for Nestin, SOX1, PAX6, and Vimentin in GMSC‐derived iNPCs determined by flow cytometry. (**F**) Western blot analysis of the expression of Nestin, SOX1, PAX6, and Vimentin GMSC‐derived iNPCs; β‐actin was used as the internal control. Abbreviations: bFGF, basic fibroblast growth factor; DAPI, 4′,6‐diamidino‐2‐phenylindole; 3D, three‐dimensional; EGF, epidermal growth factor; GMSCs, gingiva‐derived mesenchymal stem cells; iNPCs, induced neural progenitor‐like cells.

We then tested whether 3D‐spheroid neural culture could promote the induction of NPC‐like properties in GMSCs. After suspension culture for 6 days, 3D spheroids were completely dissociated into single cells (Fig. 2A), and cell viability analysis showed that more than 90% of them stayed viable (supplemental online Fig. 1G). Morphologically, these cells became smaller and relatively homogeneous in size and formed uniform and compact colonies (Fig. 2A). Upon subculture, the cells dissociated from the colonies could reform neurospheres and compact colonies under suspension and adherent culture conditions, respectively (Fig. 2B). Immunofluorescence staining showed that the majority of cells within the colonies were positive for Nestin, Sox‐1, Pax‐6, and Vimentin (Fig. 2C, 2D). Flow cytometric analysis showed that almost 100% of the induced cells were positive for Nestin and Vimentin, whereas ∼80% of the induced cells were positive for Sox‐1 and Pax‐6 (Fig. 2E). Western blot analysis confirmed the increased expression of these NSC‐related genes in induced GMSCs in comparison with noninduced and spheroid GMSCs (Fig. 2F). In addition, our results showed that 3D‐spheroid cells and iNPCs secreted a higher level of GDNF and VEGF than the parental GMSCs (supplemental online Fig. 2).

Next, we tested whether the resultant cells possessed multipotent neural differentiation capabilities. After induction for 2 weeks under neuronal or glial cell differentiation conditions 33, ∼80% of iNPCs expressed β‐tubulin III^+^, a specific marker for neuronal cells (supplemental online Fig. 3A, 3B), or S‐100β, a specific marker for Schwann cells (supplemental online Fig. 3C, 3D). The increased expression of both neuronal and Schwann cell markers in differentiated neural cells was further confirmed by Western blot (supplemental online Fig. 3E). Taken together, these results suggest that 3D‐spheroid culture enhances the induction of multipotent iNPCs directly from GMSCs, with increased capabilities to differentiate into neuronal and Schwann cells.

### Effects of GMSC‐Derived iNPCs on Axonal Regeneration at the Injury Site of the Rat Sciatic Nerve

To determine regenerative potential, GMSCs and their derivative iNPCs were transplanted to the crush‐injured site of rat sciatic nerves. Our data showed that 4 weeks after transplantation, GMSCs and iNPCs survived and engrafted at the host recipient site, specifically confined to the epineurium without directly migrating into the damaged axonal compartment (supplemental online Fig. 4B–4E). Immunofluorescence staining demonstrated that the transplanted GMSCs expressed neuronal marker β‐tubulin III (supplemental online Fig. 4B) but not Schwann cell marker S‐100β (supplemental online Fig. 4C). However, transplanted iNPCs expressed both β‐tubulin III and S‐100β (supplemental online Fig. 4D, 4E). These results suggest that iNPCs can differentiate into both neuronal and Schwann cells in the PNS environment, thus further confirming the multipotent property of iNPCs in vivo.

**Figure 3:**
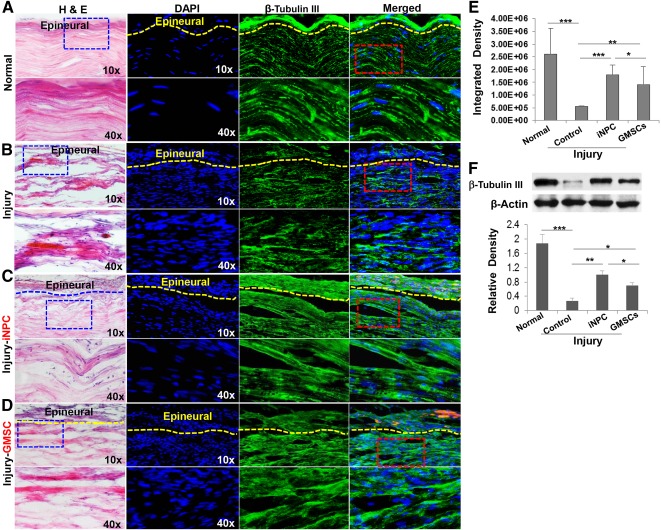
Transplantation of GMSCs and iNPCs promoted axonal regeneration of damaged sciatic nerve of rats. A cell scaffold product of GMSCs or GMSC‐derived NPC‐like cells prelabeled with PKH26 (red) and mixed with GelFoam was transplanted and wrapped around the damaged sciatic nerve of rats. Four weeks after cell transplantation, sciatic nerves were harvested and cryosections were cut for H&E staining and immunofluorescence studies on the expression of β‐tubulin III protein. Normal sciatic nerve **(A)**; injured sciatic nerve **(B)**; injured sciatic nerve with iNPC transplantation **(C)**; injured sciatic nerve with GMSC transplantation **(D)**. **(E):** Quantification of fluorescence integrated density of β‐tubulin III using ImageJ. Nuclei were counterstained with DAPI (blue). Scale bars = 100 µm (×10) or 20 µm (×40). (**F**) Expression of β‐tubulin III protein determined by Western blot analysis. ∗∗∗, *p* < .001; ∗∗, *p* < .01; ∗, *p* < .05. Abbreviations: DAPI, 4′,6‐diamidino‐2‐phenylindole; GMSC, gingiva‐derived mesenchymal stem cell; H&E, hematoxylin and eosin; iNPC, induced neural progenitor‐like cell; NPC, neural progenitor‐like cell.

Histological examination of longitudinal sections of the sciatic nerves showed that the nerve fiber displayed a more organized and aligned axonal arrangement in both GMSC and iNPC transplantation groups; in the injury control group not treated with cells, a random pattern of axonal growth was present at the injured site (Fig. 3C, 3D versus 3B, left panels). Of note, the regenerative axonal pattern of the injured sciatic nerve transplanted with iNPCs was more similar to that of the intact normal nerve (Fig. 3C versus 3A, left panels). Immunofluorescence studies showed a decreased expression of β‐tubulin III in injured nerve compared with intact normal nerve (Fig. 3B versus 3A; Fig. 3E, *p* < .001), which was further confirmed by Western blot analysis (Fig. 3F). Transplantation of GMSCs and iNPCs led to an increased expression of β‐tubulin III (Fig. 3E, 3F; Fig. 3C versus 3B, *p* < .001; Fig. 3D versus 3B, *p* < .01); iNPCs exhibited a more pronounced effect (Fig. 3C versus 3D; Fig. 3E, 3F, *p* < .05).

**Figure 4:**
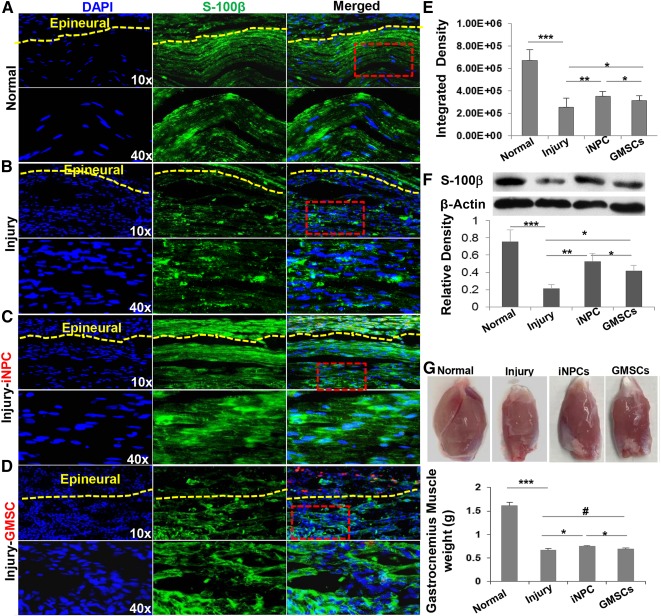
Transplantation of GMSCs and iNPCs promoted Schwann cell regeneration in the damaged sciatic nerve of rats. A cell scaffold product of GMSCs or GMSC‐derived NPC‐like cells prelabeled with PKH26 (red) and mixed with GelFoam was transplanted and wrapped around the damaged sciatic nerve of rats. Four weeks after cell transplantation, sciatic nerves were harvested and cryosections were cut for immunofluorescence studies on the protein expression of S‐100β, a marker for Schwann cells. Meanwhile, gastrocnemius muscles were harvested and weighed. Normal sciatic nerve **(A)**; injured sciatic nerve **(B)**; injured sciatic nerve with iNPC transplantation **(C)**; injured sciatic nerve with GMSC transplantation **(D)**. Nuclei were counterstained with DAPI (blue). Scale bars = 100 µm (×10) or 20 µm (×40). **(E):** Quantification of fluorescence integrated density of S‐100β using ImageJ. **(F):** Expression of S‐100β protein was determined by Western blot analysis. **(G):** Gastrocnemius muscle weight. ∗∗∗, *p* < .001; ∗∗, *p* < .01; ∗, *p* < .05; #, not significant. Abbreviations: DAPI, 4′,6‐diamidino‐2‐phenylindole; GMSC, gingiva‐derived mesenchymal stem cell; iNPC, induced neural progenitor‐like cell; NPC, neural progenitor‐like cell.

Because Schwann cells play a key role in successful guidance of axonal regeneration, we examined the expression of Schwann marker S‐100β in the sciatic nerve 4 weeks after injury and cell transplantation. In comparison with intact normal nerve, the expression of Schwann cell marker S‐100β significantly decreased in the injured nerve (Fig. 4B versus 4A; Fig. 4E, 4F, *p* < .001), whereas transplantation of GMSCs or iNPCs significantly increased S‐100β expression at the injured site (Fig. 4E, 4F; Fig. 4C versus 4B, *p* < .01; Fig. 4D versus 4B, *p* < .05). Similarly, transplantation of iNPCs showed more pronounced effects than GMSCs per se to promote S‐100β expression in the injured sciatic nerve (Fig. 4E, 4F; Fig. 4C versus 4D, *p* < .05). Functionally, we observed an overall loss of gastrocnemius muscle mass in injury and GMSC and iNPC transplantation groups at 1, 2, and 4 weeks after injury. Four weeks after injury, there was no significant difference in the average muscle weight between GMSC transplantation and the injury groups (0.640 ± 0.085 versus 0.669 ± 0.057 g; *p* = .23) (Fig. 4G). However, the average muscle weight of the iNPC transplantation group was slightly bulkier and heavier than that of the injury group (0.754 ± 0.01916 versus 0.669 ± 0.057 g, *p* = .04) and GMSC transplantation group (0.754 ± 0.01916 versus 0.640 ± 0.085 g, *p* = .016), respectively (Fig. 4G).

### Effects of GMSC‐Derived iNPCs on Axonal Regeneration Distal to the Injured Rat Sciatic Nerve

**Figure 5:**
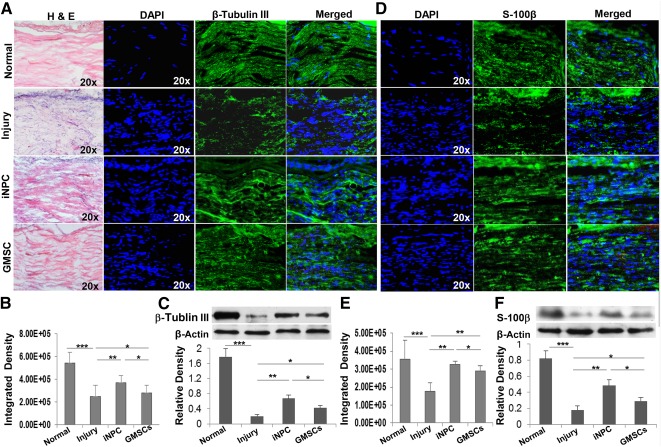
Transplantation of GMSCs/iNPCs promoted regeneration of axons and Schwann cells distal to the damaged sciatic nerves. Four weeks after cell transplantation, expression of β‐tubulin III and S‐100β proteins in segments of sciatic nerves distal to the damages was determined by immunofluorescence studies and Western blot analysis, respectively. **(A–C):** Expression of β‐tubulin III protein determined by immunofluorescence staining **(A)** and Western blot analysis **(C)**; fluorescence integrated density quantified using ImageJ **(B)**; left panel, H&E staining. **(D–F):** Expression of S‐100β determined by immunofluorescence staining **(D)** and Western blot analysis **(F)**; fluorescence integrated density quantified using ImageJ **(E)**. Nuclei were counterstained with DAPI (blue). Scale bars = 50 µm (×20). ∗∗∗, *p* < .001; ∗∗, *p* < .01; ∗, *p* < .05. Abbreviations: DAPI, 4′,6‐diamidino‐2‐phenylindole; GMSC, gingiva‐derived mesenchymal stem cell; H&E, hematoxylin and eosin; iNPC, induced neural progenitor‐like cell; NPC, neural progenitor‐like cell.

Because nerve injury often leads to Wallerian degeneration of the distal segment, we studied whether GMSCs and iNPCs can regenerate peripheral nerve distal to the injury site by examining the expression of both β‐tubulin III and S‐100β. Our results showed that transplantation of GMSCs and iNPCs significantly improved the axonal alignment of the degenerative distal nerve ends (Fig. 5A, left panels) and increased the expression of β‐tubulin III compared with the injury nerve (Fig. 5A, 5B) and Western blot analysis (Fig. 5C), respectively; the effects conferred by transplantation of iNPCs were more notable than those by GMSCs (Fig. 5B, 5C; *p* < .05). Likewise, we observed increased S‐100β expression at the distal segments of the injured nerve after transplantation of GMSCs and iNPCs (Fig. 5D–5F), whereby iNPC‐mediated effect was stronger than that mediated by GMSCs (Fig. 5E, 5F, *p* < .05). Taken together, these findings suggest that GMSC‐derived NPC‐like cells possess enhanced therapeutic potential to facilitate the repair/regeneration of injured peripheral nerves.

### GMSCs and iNPCs Regulate the Expression of Antagonistic Myelination Regulators, c‐Jun and Krox‐20/EGR2, at the Injury Site of the Rat Sciatic Nerve

**Figure 6:**
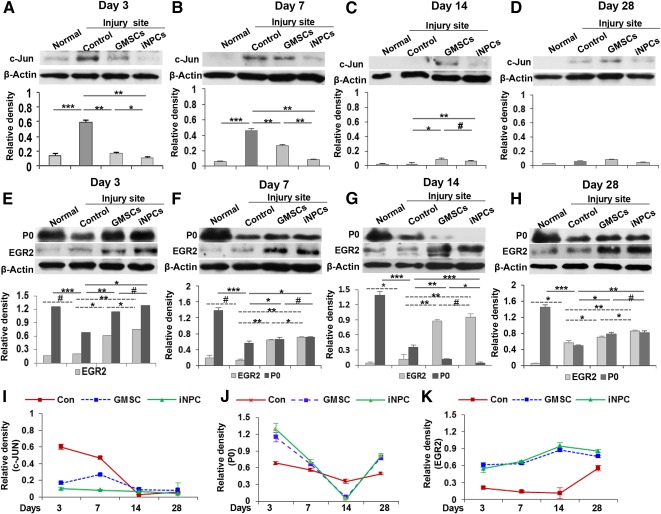
Effects of GMSC/iNPC transplantation on the expression of c‐Jun and Krox‐20/EGR2 in damaged sciatic nerves. At different time points after nerve injury and cell transplantation, sciatic nerves were harvested and tissue lysates were prepared for Western blot analysis. **(A–D):** Transplantation of GMSCs and iNPCs suppressed injury‐triggered upregulation of c‐Jun protein within 7 days after injury. **(E–H):** Effects of GMSC and iNPC transplantation on the expression of Krox‐20/EGR2 and the myelin protein P0. **(I):** Graphs show dynamic changes in the expression of c‐Jun protein summarized from Western blot analysis in Fig. 6A–6D. **(J,K):** Graphs show dynamic changes in the expression of P0 **(J)** and Krox‐20/EGR2 **(K)** proteins summarized from Western blot analysis in Fig. 6E–6H. ∗∗∗, *p* < .001; ∗∗, *p* < .01; ∗, *p* < .05; #, not significant. Abbreviations: Con, control; GMSC, gingiva‐derived mesenchymal stem cell; iNPC, induced neural progenitor‐like cell.

To further explore the potential mechanisms underlying GMSC‐ and iNPC‐mediated regenerative effects on injured peripheral nerves, we examined the dynamic changes in the expression of c‐Jun and Krox‐20/EGR2 proteins, two key cross‐antagonistic transcriptional regulators of dedifferentiation and myelination of Schwann cells, respectively 38, 39, 40, in injured rat sciatic nerves. At day 3 postinjury, the expression of c‐Jun significantly increased at the injury site; by day 7, the elevated c‐Jun started to decrease (Fig. 6A, 6B, 6I); by days 14 and 28, the levels of c‐Jun were comparable to those of normal control (Fig. 6C, 6D, 6I). However, transplantation of GMSCs or iNPCs significantly attenuated injury‐stimulated increase in c‐Jun expression within 7 days (Fig. 6A, 6B, 6I; *p* < .01), whereas iNPCs exhibited a more pronounced inhibitory effect (Fig. 6A, 6B, 6I; *p* < .05 at day 3, *p* < .01 at day 7). Fourteen days postinjury, the effects of cell transplantation on c‐Jun expression were minimal (Fig. 6C, 6D, 6I). These results suggest that GMSCs, especially GMSC‐derived iNPCs, were capable to suppress injury‐triggered upregulation in the expression of the transcription factor, c‐Jun, a dual functional regulator that positively regulates Schwann cell dedifferentiation and negatively regulates myelination 38, 39, 40.

We next analyzed the changes in the expression of myelin protein P0 (P0, *MPZ*), which constitutes a major protein component of myelin in the PNS. Within 2 weeks after injury, peripheral nerves underwent demyelination, as evidenced by a sharp decrease in the expression of P0 protein (Fig. 6E–6G, 6J; *p* < .001). Interestingly, the expression of P0 protein at the injured nerve site of the GMSC and iNPC transplantation group was relatively high in comparison with that in the injury control group within 1 week postinjury (Fig. 6E, 6F, 6J; *p* < .05). By day 14 postinjury, the levels of P0 protein in GMSC‐ and iNPC‐transplanted groups were lower than those of the injury control group (Fig. 6G, 6J; *p* < .01). Four weeks postinjury, the expression of P0 protein started to increase in injured control nerves (Fig. 6H versus 6G, second bands; *p* < .05), whereas transplantation of GMSCs and iNPCs further enhanced the expression of P0 protein (Fig. 6H, 6J; *p* < .05).

Next, we analyzed the expression of Krox‐20/EGR2, a major transcription factor that governs the expression of a set of myelination‐related genes including P0 38. Our results showed that transplantation of GMSCs and iNPCs consistently increased EGR2 protein expression at all time points after nerve injury (Fig. 6E–6H, 6K; *p* < .05), and the overall effect of iNPCs was more notable than that of GMSCs (Fig. 6E, 6F, 6H; *p* < .05). These findings suggest that at the early stages of peripheral nerve injury, GMSCs and their derivative iNPCs might delay the demyelination process and switch their functions to promote remyelination by upregulating Krox20/EGR2 protein expression at the nerve injury site.

### GMSCs and iNPCs Regulate the Expression of Antagonistic Myelination Regulators, c‐Jun and Krox‐20/EGR2, at the Distal Segment of the Injured Rat Sciatic Nerve

**Figure 7:**
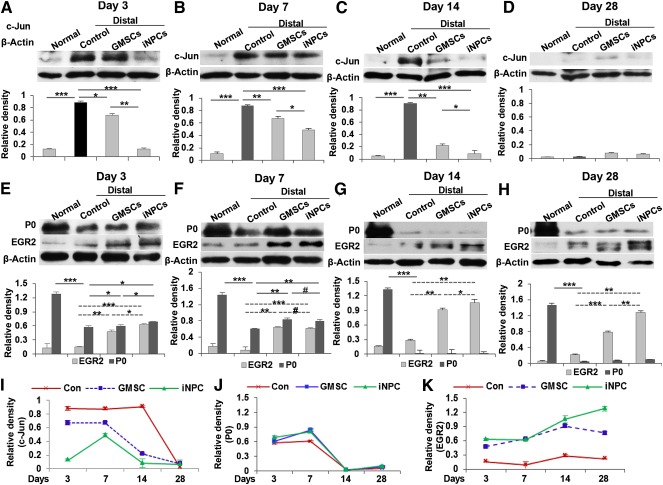
Effects of GMSC/iNPC transplantation on the expression of c‐Jun and Krox‐20/EGR2 in the distal segments of damaged sciatic nerves. At different time points after nerve injury and cell transplantation, distal segments of sciatic nerves were harvested and tissue lysates were prepared for Western blot analysis. **(A–D):** Transplantation of GMSCs and iNPCs suppressed injury‐triggered upregulation of c‐Jun protein within 14 days after injury. **(E–H):** Effects of GMSC and iNPC transplantation on the expression of Krox‐20/EGR2 and the myelin protein P0. **(I):** Graphs show dynamic changes in the expression of c‐Jun protein summarized from Western blot analysis in Fig. 6A–6D. **(J,K):** Graphs show dynamic changes in the expression of P0 **(J)** and Krox‐20/EGR2 **(K)** proteins summarized from Western blot analysis in Fig. 6E–6H. ∗∗∗, *p* < .001; ∗∗, *p* < .01; ∗, *p* < .05; #, not significant. Abbreviations: Con, control; GMSC, gingiva‐derived mesenchymal stem cell; iNPC, induced neural progenitor‐like cell.

Our results showed that transplantation of GMSCs and their derivative iNPCs promoted axonal regeneration not only at the injury site but also at the distal segment of the injured sciatic nerve (Fig. 5). Mechanistically, we sought to determine the dynamic changes in the expression of c‐Jun and EGR2 protein at the distal segment of the injured sciatic nerve after cell transplantation. Our data showed a persistent increase in the expression of c‐Jun within 14 days after injury (Fig. 7A–7C, 7I; *p* < .001). Consistent with the above findings at the injured nerve site, transplantation of GMSCs or iNPCs significantly attenuated the injury‐stimulated increase in c‐Jun expression (Fig. 7A–7C, 7I; *p* < .01), and NPC‐like cells also exhibited a more pronounced inhibitory effect at the distal segment (Fig. 7A–7C, 7I; *p* < .01 at day 3, *p* < .05 at days 7 and 14). By day 28 postinjury, the effect of cell transplantation on c‐Jun expression was minimal (Fig. 7D, 7I). These results suggest that GMSCs, especially GMSC‐derived iNPCs, could suppress injury‐triggered c‐Jun upregulation at the distal segment of the injured sciatic nerve, similar to the effects observed at the nerve injury site.

We then analyzed the changes in the expression of myelin protein P0 and the transcription factor Krox20/EGR2 at the distal segment of the injured nerve. Similar to the expression pattern of P0 protein at the injury site, a sharp decrease in the expression of this myelin protein was continuously observed at the distal segment of the injured nerve until day 14 postinjury (Fig. 7E–7G, 7J; *p* < .001); the injury‐induced decrease in P0 protein expression was attenuated in the GMSC and iNPC transplantation groups within 1 week postinjury (Fig. 7E, 7F, 7J; *p* < .05). By day 14 postinjury, the expression of P0 protein in the injury control and GMSC‐ and iNPC‐transplanted groups declined to a level that was almost undetectable (Fig. 7G, 7J). Four weeks postinjury, the expression of P0 protein started to increase in injured control nerves (Fig. 7H versus 7G, second bands; *p* < .05), whereas transplantation of GMSCs and iNPCs further enhanced its expression (Fig. 7H; *p* < .05). Similar to our findings of Krox20/EGR2 protein expression at the injury sites, transplantation of GMSCs and iNPCs consistently increased EGR2 protein expression at all time points after nerve injury (Fig. 7E–7H, 7K; *p* < .01), and iNPCs exhibited a more pronounced overall effect than GMSCs (Fig. 7E, 7G, *p* < .05; Fig. 7H, *p* < .01). Taken together, consistent with findings at the injury site, GMSCs and their derivative iNPCs might delay the demyelination process and subsequently switch to promote remyelination at the early stages of peripheral nerve injury, by upregulating Krox20/EGR2 expression at the distal segment of the injured nerve.

## Discussion

Multipotent MSCs possess potent anti‐inflammatory and immunomodulatory functions and paracrine trophic effects on tissue repair 47, 48. These unique pleiotropic properties of MSCs have made them good candidates for an allogeneic cell source in regenerative medicine. Accumulating evidence has implied that MSCs derived from tissues 9, 10, 11, particularly from dental pulp 20, 49, 50, confer beneficial effects on peripheral nerve regeneration. Mechanistically, MSC‐mediated beneficial effects on peripheral nerve regeneration have multiple facets, which may involve their multipotent transdifferentiation into different lineages of neuronal and glial cells (particularly regenerative Schwann cells) 14, 15, 19, 21, their paracrine neurotrophic 12, 20, 22 and proangiogenic 12, 16, 17 effects, and their immunomodulatory/ anti‐inflammatory and antifibrosis properties 11, 16. However, the high heterogeneity, variable neural differentiation and survival abilities, and suboptimal clinical outcomes may constitute significant hurdles for the clinical application of MSCs in peripheral nerve regeneration.

Adult neural stem cells represent a unique subpopulation of self‐renewing and multipotent neural progenitor cells 51 with inherent potential to differentiate into all types of neural cells, such as neurons, astrocytes, and oligodendrocytes. Numerous preclinical and clinical studies have demonstrated the promising efficacy of NSC‐based therapy to improve the functional recovery of damaged neural tissues under various neuropathological settings 51, 52, 53. However, difficulties in the isolation and in vitro expansion of NSCs have significantly blocked their clinical application. Most recently, several lines of evidence have demonstrated that introduction of defined sets of specific transcription factors can directly reprogram or convert differentiated somatic cells, e.g., fibroblasts, into proliferative and multipotent neural stem/progenitors 24, 25, 26, 28. Interestingly, Ring et al. 27 recently reported that self‐renewable and multipotent induced NSCs without tumorigenic potential can be generated directly from mouse and human fibroblasts reprogrammed with a single factor, SOX2, and Kim et al. 29 reported that human fibroblasts could be directly reprogrammed into induced neural crest cells by overexpression of a single transcription factor, SOX10, in combination with environmental cues, such as WNT activation. Of note, direct conversion of somatic differentiated cells to iNSCs/iNPCs by introducing exogenous factors raises clinical safety concerns, similar to iPSC technology.

Most recently, studies have demonstrated the generation of NPC‐like cells from both mouse and human somatic cells using only a cocktail of small‐molecule compounds, without the introduction of exogenous transcription factors 34. These small‐molecule compounds induce conversion toward a neural fate by inhibition of histone deacetylases, transforming growth factor‐β, and glycogen synthase kinase 3, which is accompanied by the activation of Sox2, a transcription factor that can convert somatic cells into NPCs 27. Meanwhile, NPCs can be induced directly from human adipose stem cells and human and mouse fibroblasts by simply culturing under special conditions, e.g., suspension 3D‐spheroid culture 11, 31, 32, or in defined culture media 33. The minimally manipulated approach using only small‐molecule compounds, defined culture conditions, or a combination of both without exogenous gene introduction promises a safer strategy for the generation of expandable and multipotent NPCs directly from somatic cells for autologous cell‐based therapy of neurological disorders 54.

In the present study, we show for the first time that GMSCs can be directly induced to multipotent and expandable NPC‐like cells through nongenetic approaches, and these iNPCs exhibited significantly increased secretion of the neurotrophic factor GDNF and the proangiogenic factor VEGF. More importantly, these GMSC‐derived iNPCs, after direct transplantation to the crush injury site of rat sciatic nerve, could differentiate into both neuronal and Schwann cells and exhibited potential therapeutic effects by promoting axonal regeneration at both the injury site and the distal segment of the injured nerve. In addition, we observed that transplanted GMSCs and iNPCs did not penetrate the epineurium, nor did they directly migrate to the damaged axonal compartment, suggesting that transplantation of GMSCs and iNPCs probably promote regeneration of injured nerves through their paracrine neurotrophic and proangiogenic effects. Because of their easily accessible and highly proliferative properties, neural crest origin, multipotency toward different neural lineages, and direct conversion of NPC‐like cells, GMSCs potentially serve as a superior seed cell source for autologous and/or allogeneic stem cell‐based therapy for PNI. Future studies are necessary to further explore whether cell context‐dependent induction of iNPCs requires distinct mechanisms and how to optimize the conditions to obtain adequate numbers of transplantable iNPCs in a timely manner.

In response to PNI, Schwann cells undergo reprogramming or dedifferentiation into immature precursor or progenitor‐like states 55, 56, 57, whereas myelinating Schwann cells cease to express myelin protein by downregulating the expression of Krox20/EGR2, a key transcription factor that governs myelin protein expression, while upregulating the expression of negative regulators of myelination such as the transcription factor c‐Jun 37, 38, 39. Therefore, regulation of the expression of the antagonistic c‐Jun and Krox20/EGR2 transcription factors involved in the dedifferentiation and myelination processes of Schwann cells represents a novel approach to optimize peripheral nerve regeneration. In the present study, we demonstrated for the first time to our knowledge that perineural transplantation of GMSCs, particularly GMSC‐derived iNPCs, could significantly suppress injury‐triggered increase of c‐Jun expression at both the injury site and the distal segment of the injured nerve within 1 week after injury. Meanwhile, we showed that transplantation of GMSCs and iNPCs upregulated the expression of the myelination regulator, Krox20/EGR2 protein, at both the injury site and the distal segment during nerve repair/regeneration. These findings suggest that GMSCs and iNPCs promote peripheral nerve repair/regeneration possibly by regulating both dedifferentiation and remyelination of Schwann cells in injured nerves. However, it is noteworthy that c‐Jun, in addition to its role as a negative regulator of myelination 39, 58, plays a central role in controlling both components of the Schwann cell reprogramming, dedifferentiation, and activation of the repair program, including the expression of trophic factors, adhesion molecules, formation of regeneration track, and myelin clearance 40, 58. Reduced activation of c‐Jun has been implicated in the formation of dysfunctional repair Schwann cells 40 and age‐dependent reduction in nerve regeneration 59. Therefore, further studies are warranted to identify the soluble factors and corresponding signaling pathways involved in GMSC‐ and iNPC‐mediated regulation of Schwann cell dedifferentiation and myelination.

## Author Contributions

Q.Z.: conception and design, collection and/or assembly of data, data analysis and interpretation, manuscript writing; P.N., Q.X., W.P., S.L., and A.F.: collection and/or assembly of data; A.D.L.: conception and design, manuscript writing, final approval of manuscript.

## Disclosures of Potential Conflicts of Interest

The authors indicated no potential conflicts of interest.

## Supporting information

Supporting Information

## References

[1] Zochodne DW . The challenges and beauty of peripheral nerve regrowth. J Peripher Nerv Syst 2012;17:1–18. 10.1111/j.1529-8027.2012.00378.x 22462663

[2] Angius D , Wang H , Spinner RJ et al. A systematic review of animal models used to study nerve regeneration in tissue‐engineered scaffolds. Biomaterials 2012;33:8034–8039. 22889485 10.1016/j.biomaterials.2012.07.056 PMC3472515

[3] Wood MD , Mackinnon SE . Pathways regulating modality‐specific axonal regeneration in peripheral nerve. Exp Neurol 2015;265:171–175. 25681572 10.1016/j.expneurol.2015.02.001 PMC4399493

[4] Euler de Souza Lucena E , Guzen FP , Lopes de Paiva Cavalcanti JR et al. Experimental considerations concerning the use of stem cells and tissue engineering for facial nerve regeneration: a systematic review. J Oral Maxillofac Surg 2014;72:1001–1012. 24480768 10.1016/j.joms.2013.11.006

[5] Wang Y , Li ZW , Luo M et al. Biological conduits combining bone marrow mesenchymal stem cells and extracellular matrix to treat long‐segment sciatic nerve defects. Neural Regen Res 2015;10:965–971. 26199615 10.4103/1673-5374.158362 PMC4498360

[6] Zhang Y , Zhang H , Zhang G et al. Combining acellular nerve allografts with brain‐derived neurotrophic factor transfected bone marrow mesenchymal stem cells restores sciatic nerve injury better than either intervention alone. Neural Regen Res 2014;9:1814–1819. 25422643 10.4103/1673-5374.143427 PMC4239771

[7] Liu Y , Nie L , Zhao H et al. Conserved dopamine neurotrophic factor‐transduced mesenchymal stem cells promote axon regeneration and functional recovery of injured sciatic nerve. PLoS One 2014;9:e110993. 25343619 10.1371/journal.pone.0110993 PMC4208796

[8] Tseng TC , Hsu SH . Substrate‐mediated nanoparticle/gene delivery to MSC spheroids and their applications in peripheral nerve regeneration. Biomaterials 2014;35:2630–2641. 24388817 10.1016/j.biomaterials.2013.12.021

[9] Carrier‐Ruiz A , Evaristo‐Mendonça F , Mendez‐Otero R et al. Biological behavior of mesenchymal stem cells on poly‐ε‐caprolactone filaments and a strategy for tissue engineering of segments of the peripheral nerves. Stem Cell Res Ther 2015;6:128. 26149068 10.1186/s13287-015-0121-2 PMC4522087

[10] Georgiou M , Golding JP , Loughlin AJ et al. Engineered neural tissue with aligned, differentiated adipose‐derived stem cells promotes peripheral nerve regeneration across a critical sized defect in rat sciatic nerve. Biomaterials 2015;37:242–251. 25453954 10.1016/j.biomaterials.2014.10.009

[11] Hsueh YY , Chang YJ , Huang TC et al. Functional recoveries of sciatic nerve regeneration by combining chitosan‐coated conduit and neurosphere cells induced from adipose‐derived stem cells. Biomaterials 2014;35:2234–2244. 24360575 10.1016/j.biomaterials.2013.11.081

[12] Kingham PJ , Kolar MK , Novikova LN et al. Stimulating the neurotrophic and angiogenic properties of human adipose‐derived stem cells enhances nerve repair. Stem Cells Dev 2014;23:741–754. 24124760 10.1089/scd.2013.0396

[13] Marconi S , Castiglione G , Turano E et al. Human adipose‐derived mesenchymal stem cells systemically injected promote peripheral nerve regeneration in the mouse model of sciatic crush. Tissue Eng Part A 2012;18:1264–1272. 22332955 10.1089/ten.TEA.2011.0491

[14] Tomita K , Madura T , Sakai Y et al. Glial differentiation of human adipose‐derived stem cells: implications for cell‐based transplantation therapy. Neuroscience 2013;236:55–65. 23370324 10.1016/j.neuroscience.2012.12.066

[15] Razavi S , Zarkesh‐Esfahani H , Morshed M et al. Nanobiocomposite of poly(lactide‐co‐glycolide)/chitosan electrospun scaffold can promote proliferation and transdifferentiation of Schwann‐like cells from human adipose‐derived stem cells. J Biomed Mater Res A 2015;103:2628–2634. 25614290 10.1002/jbm.a.35398

[16] Lavasani M , Thompson SD , Pollett JB et al. Human muscle‐derived stem/progenitor cells promote functional murine peripheral nerve regeneration. J Clin Invest 2014;124:1745–1756. 24642464 10.1172/JCI44071 PMC3973076

[17] Tamaki T , Hirata M , Soeda S et al. Preferential and comprehensive reconstitution of severely damaged sciatic nerve using murine skeletal muscle‐derived multipotent stem cells. PLoS One 2014;9:e91257. 24614849 10.1371/journal.pone.0091257 PMC3948784

[18] Askari N , Yaghoobi MM , Shamsara M et al. Human dental pulp stem cells differentiate into oligodendrocyte progenitors using the expression of Olig2 transcription factor. Cells Tissues Organs 2014;200:93–103. 25966902 10.1159/000381668

[19] Martens W , Sanen K , Georgiou M et al. Human dental pulp stem cells can differentiate into Schwann cells and promote and guide neurite outgrowth in an aligned tissue‐engineered collagen construct in vitro. FASEB J 2014;28:1634–1643. 24352035 10.1096/fj.13-243980 PMC4046066

[20] Yamamoto T , Osako Y , Ito M et al. Trophic effects of dental pulp stem cells on Schwann cells in peripheral nerve regeneration. Cell Transplant 2016;25:183–193. 25903498 10.3727/096368915X688074

[21] Gervois P , Struys T , Hilkens P et al. Neurogenic maturation of human dental pulp stem cells following neurosphere generation induces morphological and electrophysiological characteristics of functional neurons. Stem Cells Dev 2015;24:296–311. 25203005 10.1089/scd.2014.0117 PMC4303022

[22] Guo ZY , Sun X , Xu XL et al. Human umbilical cord mesenchymal stem cells promote peripheral nerve repair via paracrine mechanisms. Neural Regen Res 2015;10:651–658. 26170829 10.4103/1673-5374.155442 PMC4424761

[23] Chambers SM , Fasano CA , Papapetrou EP et al. Highly efficient neural conversion of human ES and iPS cells by dual inhibition of SMAD signaling. Nat Biotechnol 2009;27:275–280. 19252484 10.1038/nbt.1529 PMC2756723

[24] Kim J , Efe JA , Zhu S et al. Direct reprogramming of mouse fibroblasts to neural progenitors. Proc Natl Acad Sci USA 2011;108:7838–7843. 21521790 10.1073/pnas.1103113108 PMC3093517

[25] Lujan E , Chanda S , Ahlenius H et al. Direct conversion of mouse fibroblasts to self‐renewing, tripotent neural precursor cells. Proc Natl Acad Sci USA 2012;109:2527–2532. 22308465 10.1073/pnas.1121003109 PMC3289376

[26] Han DW , Tapia N , Hermann A et al. Direct reprogramming of fibroblasts into neural stem cells by defined factors. Cell Stem Cell 2012;10:465–472. 22445517 10.1016/j.stem.2012.02.021

[27] Ring KL , Tong LM , Balestra ME et al. Direct reprogramming of mouse and human fibroblasts into multipotent neural stem cells with a single factor. Cell Stem Cell 2012;11:100–109. 22683203 10.1016/j.stem.2012.05.018 PMC3399516

[28] Thier M , Wörsdörfer P , Lakes YB et al. Direct conversion of fibroblasts into stably expandable neural stem cells. Cell Stem Cell 2012;10:473–479. 22445518 10.1016/j.stem.2012.03.003

[29] Kim YJ , Lim H , Li Z et al. Generation of multipotent induced neural crest by direct reprogramming of human postnatal fibroblasts with a single transcription factor. Cell Stem Cell 2014;15:497–506. 25158936 10.1016/j.stem.2014.07.013

[30] Yu KR , Shin JH , Kim JJ et al. Rapid and efficient direct conversion of human adult somatic cells into neural stem cells by HMGA2/let‐7b. Cell Reports 2015 [Epub ahead of print] 10.1016/j.celrep.2014.12.038 25600877

[31] Su G , Zhao Y , Wei J et al. Direct conversion of fibroblasts into neural progenitor‐like cells by forced growth into 3D spheres on low attachment surfaces. Biomaterials 2013;34:5897–5906. 23680365 10.1016/j.biomaterials.2013.04.040

[32] Mirakhori F , Zeynali B , Kiani S et al. Brief azacytidine step allows the conversion of suspension human fibroblasts into neural progenitor‐like cells. Cell J 2015;17:153–158. 25870845 10.22074/cellj.2015.522 PMC4393664

[33] Feng N , Han Q , Li J et al. Generation of highly purified neural stem cells from human adipose‐derived mesenchymal stem cells by Sox1 activation. Stem Cells Dev 2014;23:515–529. 24138016 10.1089/scd.2013.0263 PMC3929335

[34] Cheng L , Hu W , Qiu B et al. Generation of neural progenitor cells by chemical cocktails and hypoxia. Cell Res 2014;24:665–679. 24638034 10.1038/cr.2014.32 PMC4042166

[35] Leite C , Silva NT , Mendes S et al. Differentiation of human umbilical cord matrix mesenchymal stem cells into neural‐like progenitor cells and maturation into an oligodendroglial‐like lineage. PLoS One 2014;9:e111059. 25357129 10.1371/journal.pone.0111059 PMC4214693

[36] Gaudet AD , Popovich PG , Ramer MS . Wallerian degeneration: Gaining perspective on inflammatory events after peripheral nerve injury. J Neuroinflammation 2011;8:110. 21878126 10.1186/1742-2094-8-110 PMC3180276

[37] Kim HA , Mindos T , Parkinson DB . Plastic fantastic: Schwann cells and repair of the peripheral nervous system. Stem Cells Translational Medicine 2013;2:553–557. 23817134 10.5966/sctm.2013-0011 PMC3726134

[38] Pereira JA , Lebrun‐Julien F , Suter U . Molecular mechanisms regulating myelination in the peripheral nervous system. Trends Neurosci 2012;35:123–134. 22192173 10.1016/j.tins.2011.11.006

[39] Parkinson DB , Bhaskaran A , Arthur‐Farraj P et al. c‐Jun is a negative regulator of myelination. J Cell Biol 2008;181:625–637. 18490512 10.1083/jcb.200803013 PMC2386103

[40] Arthur‐Farraj PJ , Latouche M , Wilton DK et al. c‐Jun reprograms Schwann cells of injured nerves to generate a repair cell essential for regeneration. Neuron 2012;75:633–647. 22920255 10.1016/j.neuron.2012.06.021 PMC3657176

[41] Zhang Q , Shi S , Liu Y et al. Mesenchymal stem cells derived from human gingiva are capable of immunomodulatory functions and ameliorate inflammation‐related tissue destruction in experimental colitis. J Immunol 2009;183:7787–7798. 19923445 10.4049/jimmunol.0902318 PMC2881945

[42] Zhang QZ , Nguyen AL , Yu WH et al. Human oral mucosa and gingiva: A unique reservoir for mesenchymal stem cells. J Dent Res 2012;91:1011–1018. 22988012 10.1177/0022034512461016 PMC3490281

[43] Zhang QZ , Su WR , Shi SH et al. Human gingiva‐derived mesenchymal stem cells elicit polarization of m2 macrophages and enhance cutaneous wound healing. Stem Cells 2010;28:1856–1868. 20734355 10.1002/stem.503 PMC3114043

[44] Xu X , Chen C , Akiyama K et al. Gingivae contain neural‐crest‐ and mesoderm‐derived mesenchymal stem cells. J Dent Res 2013;92:825–832. 23867762 10.1177/0022034513497961 PMC3744273

[45] Sung MA , Jung HJ , Lee JW et al. Human umbilical cord blood‐derived mesenchymal stem cells promote regeneration of crush‐injured rat sciatic nerves. Neural Regen Res 2012;7:2018–2027. 25624833 10.3969/j.issn.1673-5374.2012.26.003 PMC4296421

[46] Potapova TA , Sivakumar S , Flynn JN et al. Mitotic progression becomes irreversible in prometaphase and collapses when Wee1 and Cdc25 are inhibited. Mol Biol Cell 2011;22:1191–1206. 21325631 10.1091/mbc.E10-07-0599 PMC3078080

[47] Trounson A , McDonald C . Stem cell therapies in clinical trials: Progress and challenges. Cell Stem Cell 2015;17:11–22. 26140604 10.1016/j.stem.2015.06.007

[48] Bianco P . “Mesenchymal” stem cells. Annu Rev Cell Dev Biol 2014;30:677–704. 25150008 10.1146/annurev-cellbio-100913-013132

[49] Sakai K , Yamamoto A , Matsubara K et al. Human dental pulp‐derived stem cells promote locomotor recovery after complete transection of the rat spinal cord by multiple neuro‐regenerative mechanisms. J Clin Invest 2012;122:80–90. 22133879 10.1172/JCI59251 PMC3248299

[50] Mead B , Logan A , Berry M et al. Intravitreally transplanted dental pulp stem cells promote neuroprotection and axon regeneration of retinal ganglion cells after optic nerve injury. Invest Ophthalmol Vis Sci 2013;54:7544–7556. 24150755 10.1167/iovs.13-13045

[51] Miller RH . The promise of stem cells for neural repair. Brain Res 2006;1091:258–264. 16563359 10.1016/j.brainres.2006.01.073

[52] Nam H , Lee KH , Nam DH et al. Adult human neural stem cell therapeutics: Current developmental status and prospect. World J Stem Cells 2015;7:126–136. 25621112 10.4252/wjsc.v7.i1.126 PMC4300923

[53] Martino G , Pluchino S . The therapeutic potential of neural stem cells. Nat Rev Neurosci 2006;7:395–406. 16760919 10.1038/nrn1908

[54] Mirakhori F , Zeynali B , Salekdeh GH et al. Induced neural lineage cells as repair kits: So close, yet so far away. J Cell Physiol 2014;229:728–742. 24242901 10.1002/jcp.24509

[55] Jang SY , Shin YK , Park SY et al. Autophagic myelin destruction by Schwann cells during Wallerian degeneration and segmental demyelination. Glia 2016;64:730–742. 26712109 10.1002/glia.22957

[56] Gomez‐Sanchez JA , Carty L , Iruarrizaga‐Lejarreta M et al. Schwann cell autophagy, myelinophagy, initiates myelin clearance from injured nerves. J Cell Biol 2015;210:153–168. 26150392 10.1083/jcb.201503019 PMC4494002

[57] DeFrancesco‐Lisowitz A , Lindborg JA , Niemi JP et al. The neuroimmunology of degeneration and regeneration in the peripheral nervous system. Neuroscience 2015;302:174–203. 25242643 10.1016/j.neuroscience.2014.09.027 PMC4366367

[58] Jessen KR , Mirsky R . The repair Schwann cell and its function in regenerating nerves. J Physiol 2016;594:3521–3531. 26864683 10.1113/JP270874 PMC4929314

[59] Painter MW , Brosius Lutz A , Cheng YC et al. Diminished Schwann cell repair responses underlie age‐associated impaired axonal regeneration. Neuron 2014;83:331–343. 25033179 10.1016/j.neuron.2014.06.016 PMC4106408

