# Supplementary material for: Neural Progenitor‐Like Cells Induced from Human Gingiva‐Derived Mesenchymal Stem Cells Regulate Myelination of Schwann Cells in Rat Sciatic Nerve Regeneration
Source: Stem Cells Transl Med. 2016 Sep 7;6(2):458–70. doi: 10.5966/sctm.2016-0177 (PMC5442816; doi:10.5966/sctm.2016-0177)
Supplement: Supplementary file 1 — Supporting Information [file SCT3-6-458-s001.pdf]

Figure S1-R1

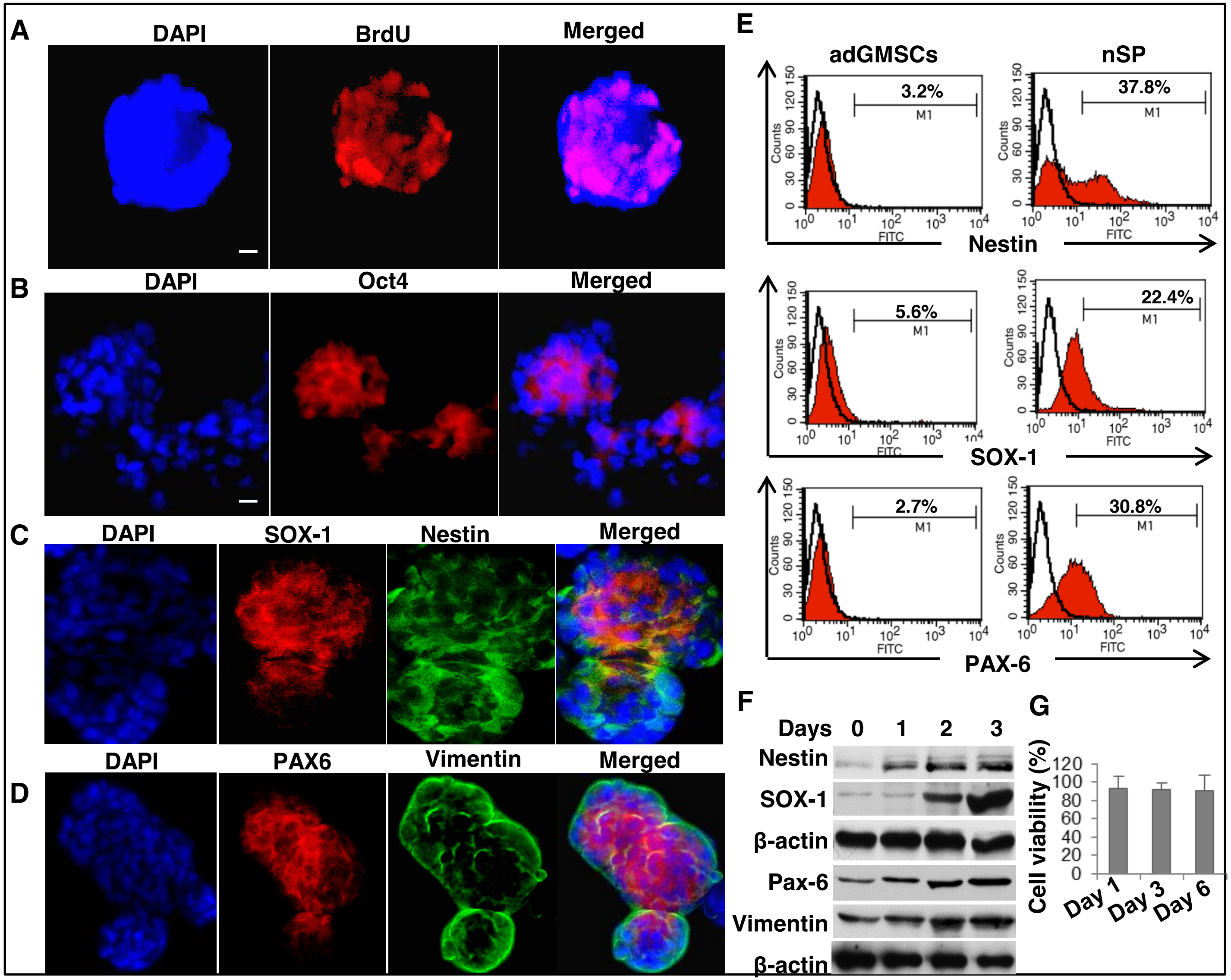

A
DAPI
BrdU
Merged
B
DAPI
Oct4
Merged
C
DAPI
SOX-1
Nestin
Merged
D
DAPI
PAX6
Vimentin
Merged
E
adGMSCs
nSP
3.2%
37.8%
M1
Counts
FITC
Nestin
5.6%
22.4%
SOX-1
2.7%
30.8%
PAX-6
F
Days 0 1 2 3
Nestin
SOX-1
β-actin
Pax-6
Vimentin
β-actin
G
Cell viability (%)
Day 1
Day 3
Day 6

Figure S2-R1

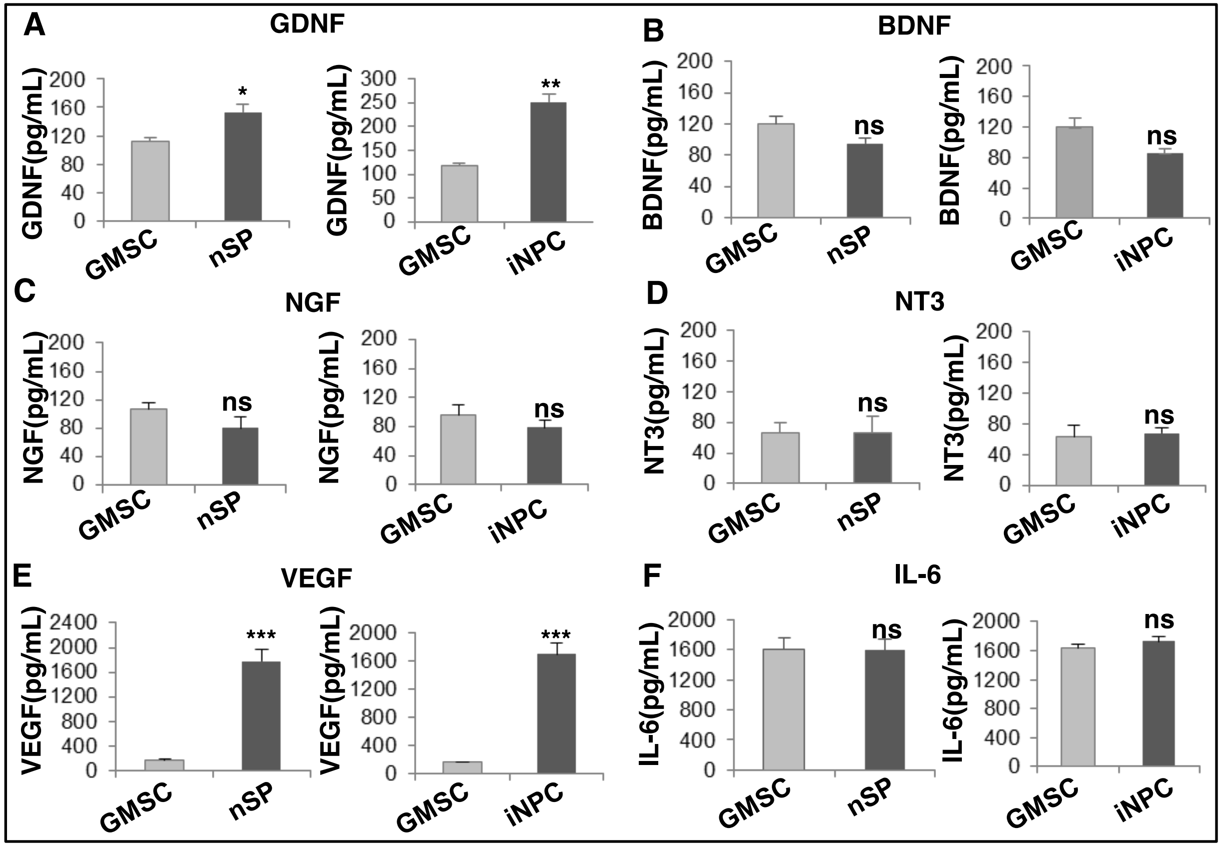

A
GDNF
GDNF(pg/mL)
200
160
120
80
40
0
*
GMSC
nSP
300
250
200
150
100
50
0
**
GMSC
iNPC
B
BDNF
BDNF(pg/mL)
ns
GMSC
nSP
GMSC
iNPC
C
NGF
NGF(pg/mL)
ns
GMSC
nSP
GMSC
iNPC
D
NT3
NT3(pg/mL)
ns
GMSC
nSP
GMSC
iNPC
E
VEGF
VEGF(pg/mL)
2400
2000
1600
1200
800
400
0
***
GMSC
nSP
GMSC
iNPC
F
IL-6
IL-6(pg/mL)
ns
GMSC
nSP
GMSC
iNPC

Figure S3

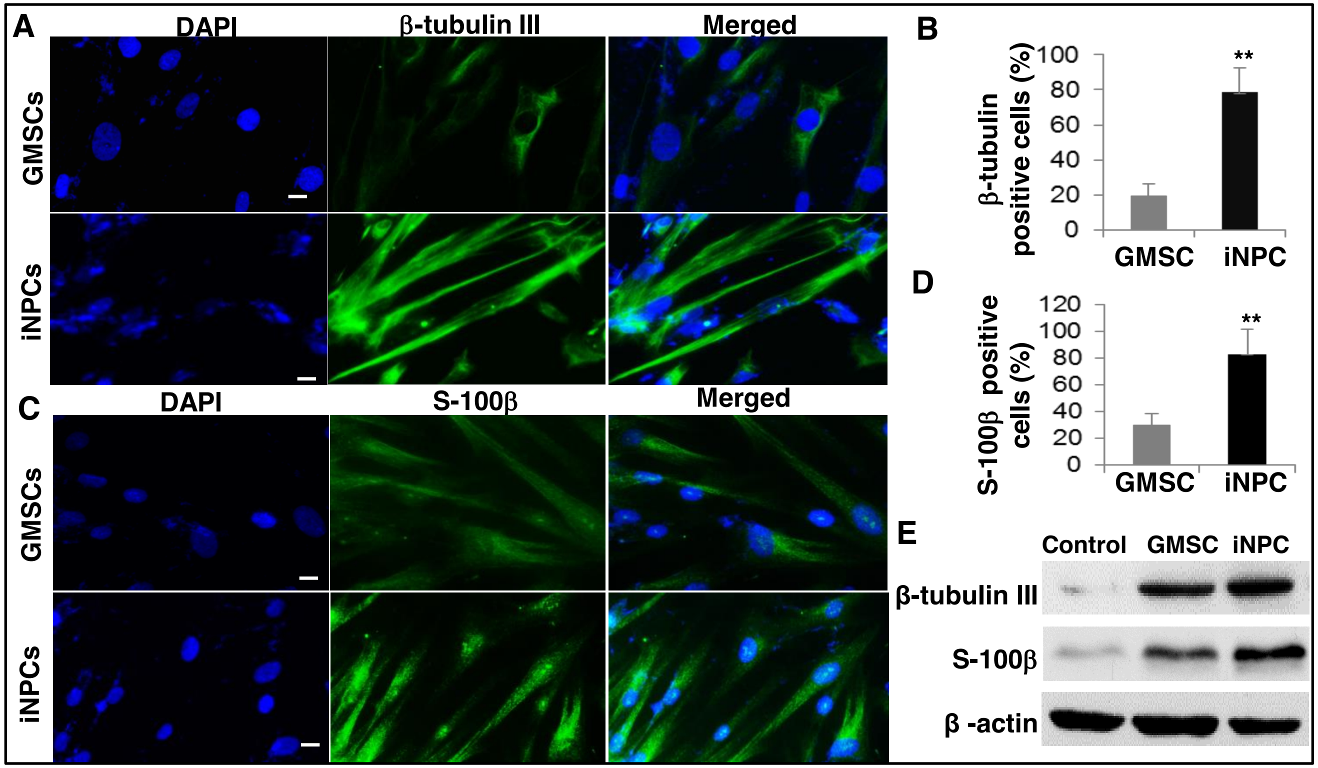

A
DAPI
β-tubulin III
Merged
GMSCs
iNPCs
B
β-tubulin positive cells (%)
100
80
60
40
20
0
**
GMSC
iNPC
C
DAPI
S-100β
Merged
GMSCs
iNPCs
D
S-100β positive cells (%)
120
100
80
60
40
20
0
**
GMSC
iNPC
E
Control
GMSC
iNPC
β-tubulin III
S-100β
β -actin

Figure S4-R1

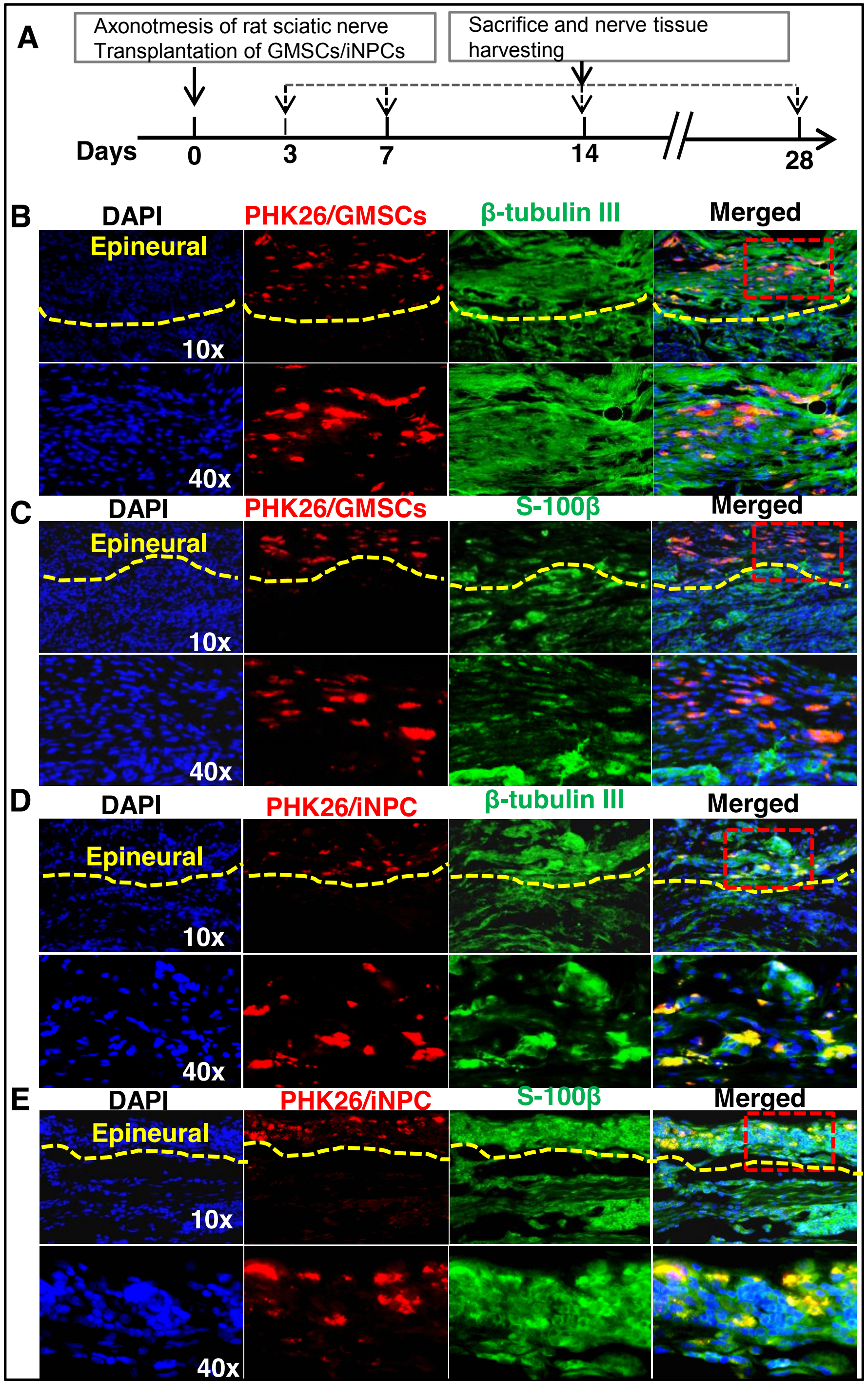

A
Axonotmesis of rat sciatic nerve
Transplantation of GMSCs/iNPCs
Sacrifice and nerve tissue harvesting
Days
0
3
7
14
28
B
DAPI
PHK26/GMSCs
β-tubulin III
Merged
Epineural
10x
40x
C
DAPI
PHK26/GMSCs
S-100β
Merged
Epineural
10x
40x
D
DAPI
PHK26/iNPC
β-tubulin III
Merged
Epineural
10x
40x
E
DAPI
PHK26/iNPC
S-100β
Merged
Epineural
10x
40x

Fig. S1. Increased expression of neural stem cell-related genes in spheroid GMSCs. GMSCs were cultured in neurobasal medium supplemented with 1% N2, 2% B27, 20 ng/mL EGF, and 20 ng/mL bFGF, under suspension culture condition for 3 days. (**A-D**) Immunofluorescence studies on the expression of Nestin, SOX1, PAX6, and Vimentin, in spheroid GMSCs. Nuclei were counterstained with 4’, 6-diamidino-2-phenylindole (DAPI; blue). Scale bars, 20μm (40×). (**E**) The percentage of cells positive for Nestin, SOX1, and PAX6 in GMSCs cultured under either regular adherent (adGMSC) or suspension spheroid (nSP) culture conditions, as determined by flow cytometry. (**F**) Western blot analysis of the expression of Nestin, SOX1, PAX6, and Vimentin in cells following spheroid culture for different days, while β-actin was used as the internal control. (**G**) Following spheroid culture for different days, the cell viability of dissociated single cells was determined by trypan blue staining. Abbreviations: BrdU, 5-bromo-2'-deoxyuridine; adGMSC, adherent GMSCs; nSP, neural spheroid cells.

Fig. S2. ELISA analysis of neurotrophic factors and cytokines secreted by GMSCs, neural spheroid cells and iNPCs. GMSCs, neural spheroids and iNPCs ($2x10^5$/well) were seeded in 6-well plates and cultured under distinct conditions for 3 days and the conditioned media were collected for ELISA analysis on the secretion of various neurotrophic factors, GDNF (**A**), BDNF (**B**), NGF (**C**), NT3 (**D**), and cytokines VEGF (**E**) and IL-6 (**F**). ***P<0.001; **P<0.01; *P<0.05; ns, no significance.

Fig. S3. Differentiation of iNPCs into neuronal and glial cells. GMSCs and iNPC were cultured under neuronal or glial cell induction conditions for 14 days**.** The expression of the neuron marker, β-tubulin III (**A**), and the Schwann cell marker, S-100β (**C**), were determined by immunofluorescence staining. Nuclei were counterstained with 4’, 6-diamidino-2-phenylindole (DAPI; blue). The images shown are representative of a randomly selected field from three independent cultures. (**B**, **D**) For semi-quantitative analysis, cells with positive signals in at least six random high-power fields (HPF) were visualized, counted and expressed as the percentage of total DAPI-positive cells. The data were representative of results from three independent studies. Scale bars, 20μm (40×). **P<0.01. (**E**): GMSCs and iNPC were cultured under neuronal or glial cell induction conditions for 14 days, and the expression of β-tubulin III and S-100β was determined by Western blot analysis, while the non-differentiated GMSCs were used as control.

Fig. S4. Transplanted GMSCs and iNPCs differentiated into neuronal and Schwann cells *in vivo*. (**A**) A diagram showing the timelines for nerve injury and cell transplantation. A cell scaffold product of GMSCs or GMSC-derived NPC-like cells pre-labeled with PKH26 (Red) and mixed with GelFoam was transplanted and wrapped around the damaged sciatic nerve of rats induced by axonotmesis crush injury. (**B, C**) Immunofluorescence studies on the expression of β-tubulin III (**B**) or S-100β (**C**) in GMSCs following transplantation for 4 weeks. (**D, E**) Immunofluorescence studies on the expression of β-tubulin III (**D**) or S-100β (**E**) in iNPCs following transplantation for 4 weeks. Nuclei were counterstained with 4', 6-diamidino-2-phenylindole (DAPI; blue). Scale bars, 100 µm (10×) or 20µm (40×). Abbreviations: iNPCs, induced neural progenitor-like cells.
